# Effect of Native Oxide Layer on Mechanochemical Reaction at the GaN–Al_2_O_3_ Interface

**DOI:** 10.3389/fchem.2021.672240

**Published:** 2021-05-04

**Authors:** Jian Guo, Chen Xiao, Jian Gao, Jinwei Liu, Lei Chen, Linmao Qian

**Affiliations:** ^1^School of Mechanical Engineering, University of South China, Hengyang, China; ^2^State Key Laboratory of Traction Power, Tribology Research Institute, Southwest Jiaotong University, Chengdu, China; ^3^Advanced Research Center for Nanolithography, Amsterdam, Netherlands

**Keywords:** ambient humidity, Arrhenius-type kinetics model, GaN–Al_2_O_3_ interface, mechanochemical removal, native oxide layer

## Abstract

Mechanochemical reactions at the gallium nitride-alumina (GaN–Al_2_O_3_) interface at nanoscale offer a significant beneficial reference for the high-efficiency and low-destruction ultra-precision machining on GaN surface. Here, the mechanochemical reactions on oxide-free and oxidized GaN surfaces rubbed by the Al_2_O_3_ nanoasperity as a function of the ambient humidity were studied. Experimental results reveal that oxidized GaN exhibits a higher mechanochemical removal rate than that of oxide-free GaN over the relative humidity range of 3–80%. The mechanical activation in the mechanochemical reactions at the GaN–Al_2_O_3_ interface is well-described by the mechanically-assisted Arrhenius-type kinetics model. The analysis indicates that less external mechanical activation energy is required to initiate the mechanochemical atomic attrition on the oxidized GaN surface compared with the oxide-free GaN surface. These results may not only gain a deep understanding of the mechanochemical removal mechanism of GaN but also provide the basic knowledge for the optimization of the oxidation-assisted ultra-precision machining.

## Introduction

Because of the excellent performances in the case of wide direct bandgap, high heat capacity and thermal conductivity, low dielectric constant, and high breakdown voltage, gallium nitride (GaN) materials have aroused great interests in the wide application prospects of optoelectronics and microelectronic, such as high-brightness/efficiency light-emitting diodes, high-frequency/power/temperature transistors, short-wavelength emitters/detectors, etc. (Tsao et al., 2014; Pust et al., 2015; Glavin et al., 2017; Lev et al., 2018; Meneghesso et al., 2018; Sarangadharan et al., 2018). The chemical mechanical polishing (CMP) technique is a prerequisite to obtaining high-quality wafers for various applications in GaN-based devices as mentioned above. By utilizing the coupling effect of external mechanical interaction and chemical reaction, CMP can realize vital planarization and minimize the surface/subsurface damages to improve the working stability and service lifetime of electronics (Aida et al., 2012; Gong et al., 2017; Shi et al., 2017; Kubota and Iwakiri, 2019). Due to the repeated action of multiple abrasive particles and the small contact area in the ultra-precision machining, the instantaneous removal of the material makes the actual reaction process unable to be real-time monitored and quantitatively characterized. To reveal the mechanochemical removal mechanism during the CMP process of GaN surface, nanowear test under the single-point contact situation is essential to simulate the actual machining process through controlling the parameters, such as load, speed, processing time, and processing trajectory.

Tribological properties are sensitive not only to the intrinsic factors (e.g., hardness, elastic modulus, chemical activity, and bonding energy) but also to the extrinsic factors (e.g., humidity, temperature, load/contact pressure, velocity/time) (Hayashi et al., 2007; Zhao and Lu, 2013; Asghar et al., 2014; Yu et al., 2016; Zeng et al., 2016, 2017, 2018a; Zhang et al., 2018; Carlton et al., 2020). Previous studies have indicated that with the assistance of the chemically active counter-surface and ambient medium, the mechanochemical material removal can occur under the pure elastic contact situation (the contact pressure is far below the plastic yield of the substrate). Such material removal is dominated by the interfacial mechanochemical reaction involving the formation of interfacial bonding bridges and the rupture of chemical bonds on the substrate (Chen et al., 2014; Xiao et al., 2017a). Hereinto, the external mechanical energy originating from the compressive and shear forces of chemically active counter-surface weakens the energy barrier of the reaction kinetics to activate the mechanochemical atomic attrition. As a chemical means or process initiated from the contact interface, the potentiality of mechanochemical reactions can be influenced by the surface states. Inevitable surface oxidation in the manufacturing process induces changes in surface wettability, mechanical property, and atomic structure, leading to complex and uncertain effects (Xiao et al., 2020; Gao et al., 2021). However, limited literature has been reported on the influence of surface oxidization on mechanochemical removal of GaN surface.

In this work, the material removal behaviors of the oxide-free and oxidized GaN surfaces against the Al_2_O_3_ microsphere are investigated at the nanoscale. Experimental results show that the mechanochemical reactions at the GaN–Al_2_O_3_ interface can be facilitated by the topmost oxidized surface structure. The mechanical activation in the mechanochemical removal of the oxide-free and oxidized GaN surfaces can be well-described with the mechanically-assisted Arrhenius-type kinetics model.

## Materials and Methods

### Material Preparation

**Figure 1:**
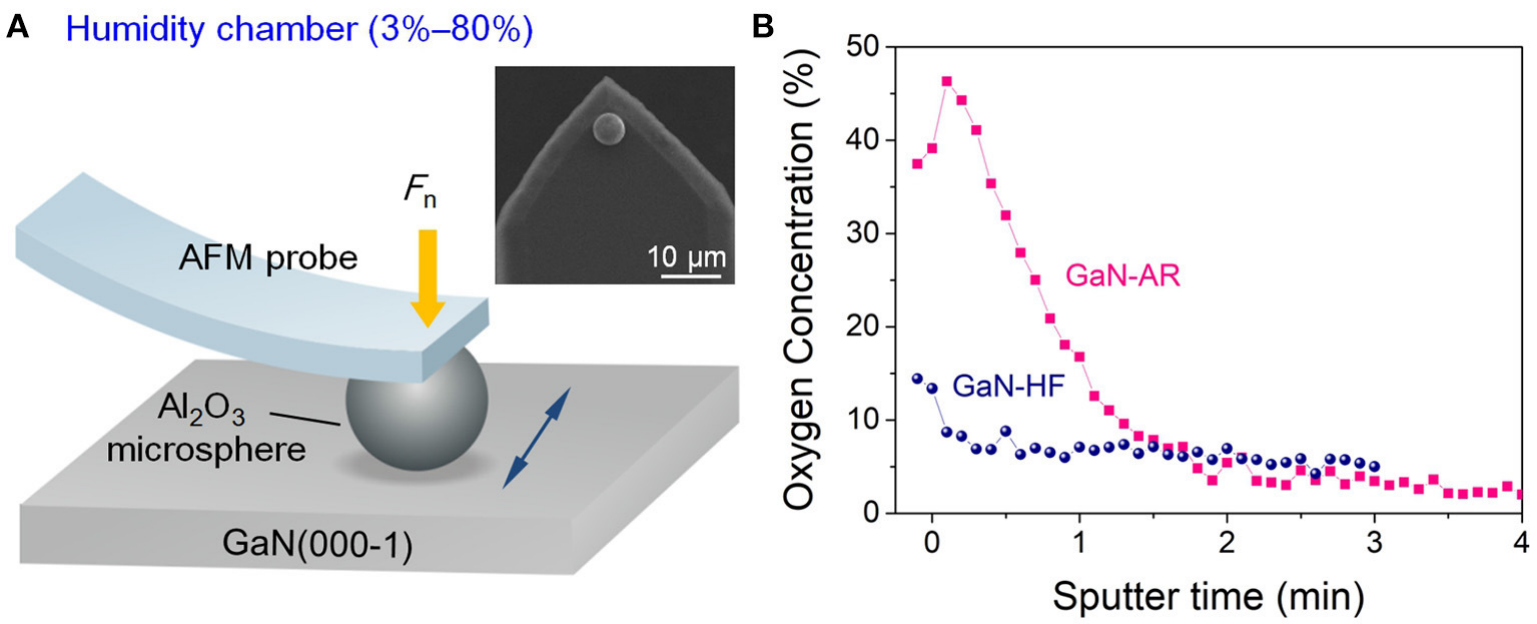
Schematic drawing of nanowear tests on the N-faced GaN surface. **(A)** Mechanochemical removal test on GaN surface with the AFM probes in a home-built humidity-control system. Insets show the SEM images of the Al_2_O_3_ microsphere. **(B)** Oxygen concentration as a function of sputter time for GaN-AR and GaN-HF surfaces.

The undoped *c*-plane bulk GaN wafers (Heifei Crystal Technical Material Co., Ltd, China) prepared using the hydride vapor phase epitaxy technique were used for our experiments (Figure 1A). These GaN samples were subjected to double-sided polishing following the universal CMP protocol. Due to the continuous oxidation during the prolonged exposure to ambient air containing oxygen and water molecules, the GaN sample surface was covered with a native oxide layer of ~4 nm according to variation of oxygen concentration with sputter depth by using the Auger electron spectrum (AES, PHI-700, ULVAC-PHI, Inc., Kanagawa, Japan), as shown in Figure 1B. This sample was denoted as “GaN-AR.” Note that the sputtering rate was calibrated using the thermally oxidized SiO_2_ standard sample, hence the thickness of oxide layer on GaN surface here is an estimated value. After immersing the GaN-AR in 3 wt.% hydrofluoric acid (HF) solution for 3 min, the native oxide layer on GaN surface can be almost completely removed, such GaN sample (oxide-free) was denoted as “GaN-HF.” The root-mean-square roughness values of GaN-AR and GaN-HF surfaces were estimated at <0.5 nm over an area of 10 × 10 μm using an atomic force microscope (AFM, SPA-300HV Probe Station, Seiko, Japan). Before nanowear tests, all the samples were ultrasonic cleaned in acetone, ethanol and rinsed with deionized water in sequence, and then dried using pure N_2_ gas.

### Nanowear and Nanoindentation Tests

To eliminate the influence of complex multi-asperities contact at the tribological interface and precisely control the contact pressure, sliding velocity, and environmental media, the nanowear tests were performed by an AFM equipped with a home-built humidity-control system (Figure 1A). Detailed information about the humidity-control system could be found in previous literature (Xiao et al., 2017b). Two kinds of AFM probes, one was with a conic diamond tip with a nominal radius *R* of ~20 nm attached to a cantilever with a spring constant *k* of ~100 N/m, and the other with a spherical Al_2_O_3_ tip (or being called as Al_2_O_3_ microsphere) with *R* of ~2.5 μm attached to a cantilever with *k* of ~19.8 N/m, were used in nanowear tests to compare the mechanical removal and mechanochemical removal of GaN-AR and GaN-HF surfaces, respectively. Reciprocating nanowear tests on GaN-AR and GaN-HF surfaces against Al_2_O_3_ microsphere were performed with a sliding amplitude of 1 μm under the following conditions: applied load (*F*_n_) ranged within 0.5–5 μN, sliding speed (*v*) varied within 0.1–1,000 μm/s, relative humidity (RH) varied within 3–80%, and ambient temperature was maintained at 25 ± 2°C. All the removal tracks were scanned by using a sharp Si_3_N_4_ probe (MLCT, Bruker, MA, USA) with *R* of ~20 nm and *k* of ~0.1 N/m in a vacuum condition of 10^−4^ torr. Nanoindentation tests on GaN-HF and GaN-AR surfaces were performed using nanomechanical test instruments (TI750, Hysitron, Bruker, MA, USA) with a spherical diamond nanoindenter with a radius of 1 μm.

### AES and Selected-Area X-ray Photoelectron Spectroscopy Characterization

The oxygen concentration varied with the sputter depth for GaN-AR and GaN-HF substrates was analyzed by AES using a coaxial electron gun and cylindrical mirror analyzer. To investigate the material removal mechanism of GaN surfaces against Al_2_O_3_ counter-surface, the chemical states and elemental compositions of pristine surface, and wear debris were analyzed by SAXPS (PHI VersaProbe III, Physical Electronics, Inc., MN, USA) with a monochromatic X-ray source and a lateral resolution <20 μm. Dual beams (electron and ion) were used for XPS characterization to avoid electrical charging of the detected sample. The wear debris for the XPS characterization was produced by an Al_2_O_3_ sphere with a radius of 1.5 mm using a universal micro-tribotester (UMT-5, Bruker, MA, USA).

## Results and Discussion

### Nanowear Behaviors of GaN-HF and GaN-AR Surfaces Against Al_2_O_3_ Tip

**Figure 2:**
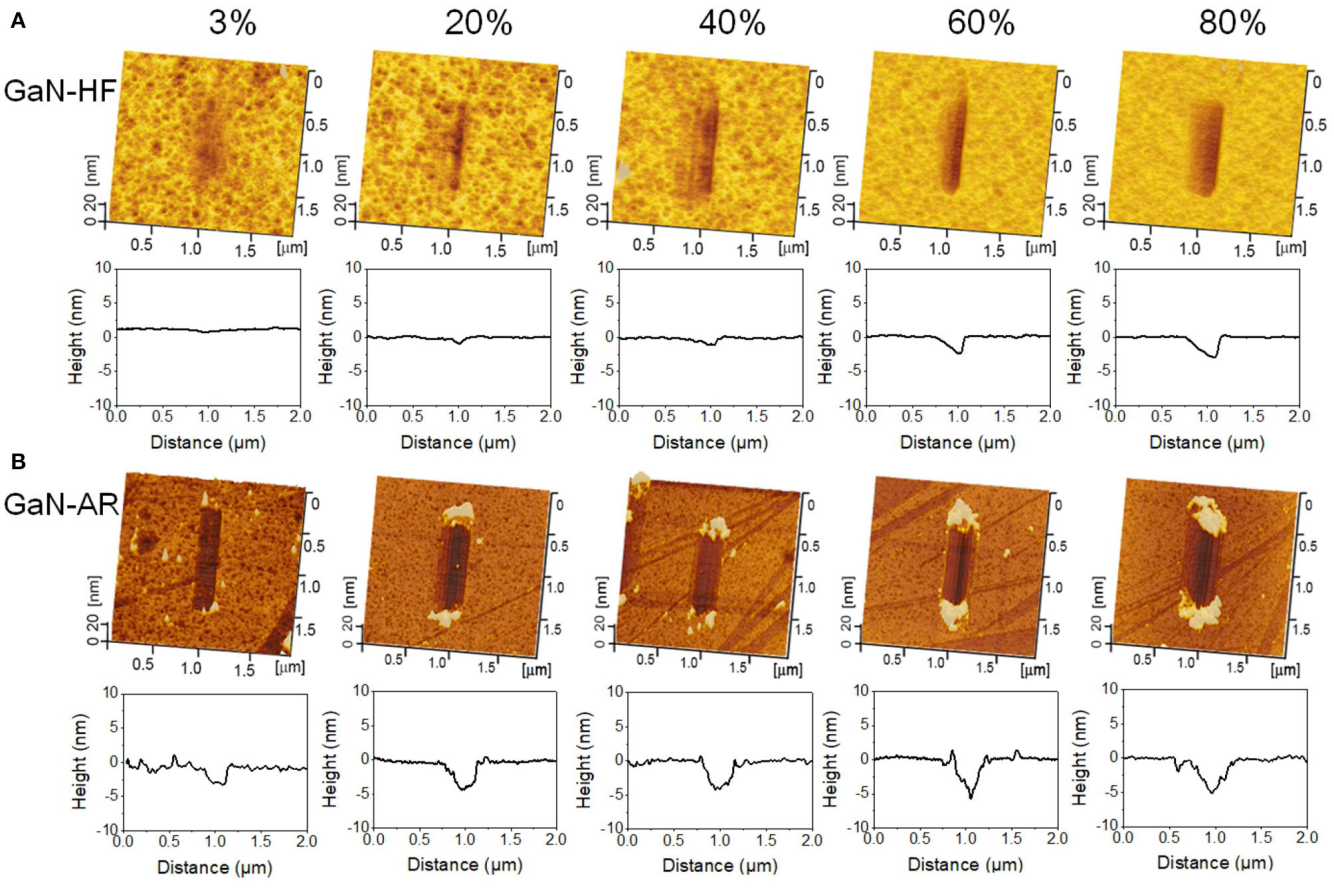
AFM images and average cross-sectional profiles of the wear tracks on **(A)** GaN-HF and **(B)** GaN-AR surfaces produced by sliding the Al_2_O_3_ tip at different RHs under the conditions of *F*_n_ = 4 μN, *v* = 2 μm/s, *N* = 2000, and RH = 3–80%.

**Figure 3:**
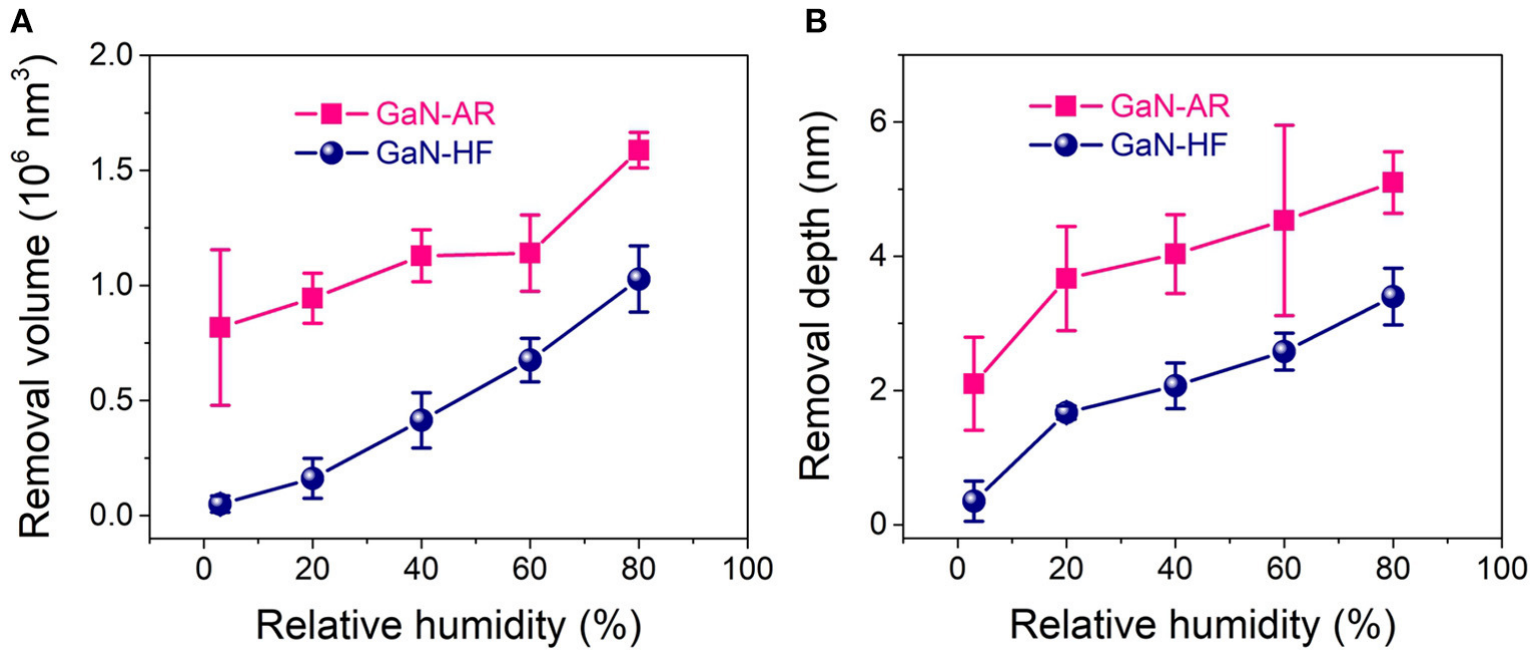
**(A)** Removal volume vs. relative humidity; **(B)** Removal depth vs. relative humidity.

Nanowear tests were performed in the ambient environment with the RH range of 3–80% to reveal the mechanochemical removal behaviors of GaN-HF and GaN-AR surfaces against the Al_2_O_3_ microsphere. Figures 2A,B, respectively, display the AFM images and the average cross-sectional profiles of wear tracks on GaN-HF and GaN-AR surfaces for different RHs. The removal volume and depth as a function of relative humidity were plotted in Figure 3. Slight wear with a depth of ~0.2 nm occurred on the GaN-HF surface in dry N_2_ condition and the removal volume was dramatically increased from 10^5^ nm^3^ at 3% RH to 10^6^ nm^3^ at 80% RH. Based on the Derjaguin-Muller-Toporov (DMT) contact theory, 4 μN normal load in the reciprocating wear process provides a maximum contact pressure of ~1.6 GPa, which is far below the yield strength of crystal GaN material (~20 GPa) (Schwarz, 2003). In this case, the material removal of GaN-HF substrate is not caused by the mechanical action of the Al_2_O_3_ counter-surface, but by the water-participated interfacial mechanochemical reaction that is highly humidity-dependent (Guo et al., 2021a).

Moreover, the experimental result of the GaN-AR surface also demonstrated a positive excitation relationship between material removal and ambient humidity. As the RH increased from 3 to 80%, the wear volume increased by 93% (from 0.8 × 10^6^ to 1.5 × 10^6^ nm^3^), but it was far below the increase rate of 10 times for the GaN-HF case. Even at 3% RH, the wear track with a depth of 2 nm was observed on the GaN-AR surface, and its removal depth is much higher than that of the wear track produced on the GaN-HF surface. Given that those tests on GaN-HF and GaN-AR surfaces were conducted under the same loading conditions, the differences in wear should be mainly attributed to the covered oxide layer on the GaN-AR surface with different mechanical and chemical properties from monocrystalline GaN. Further discussion can be found in section Influencing mechanism of oxide layer on the mechanochemical removal of GaN surface. Besides, the wear debris on the GaN-HF surface could be washed out easily by ultrasonic cleaning; By contrast, the wear debris on the GaN-AR surface was pushed to both ends of the scratching track by the tip and piles up firmly (Yu et al., 2016). The state of wear debris on the substrate may be strongly dependent on the surface hydrophilicity of the material and the environmental humidity (Zhang et al., 2019).

### Nanowear Behaviors of GaN-HF and GaN-AR Surfaces Against Diamond Tip

**Figure 4:**
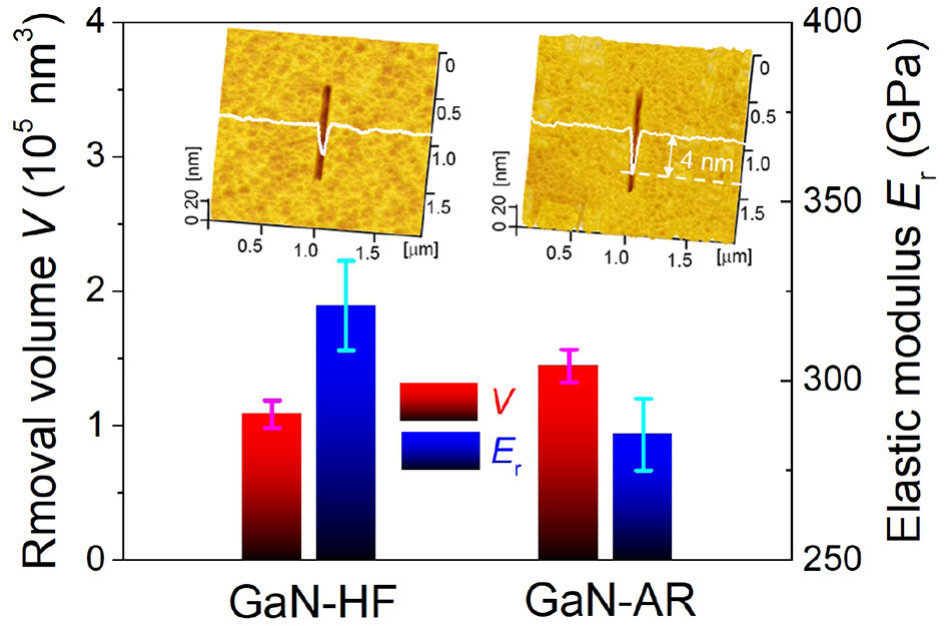
Correlation of elastic modulus and removal volume on GaN-HF and GaN-AR surfaces. These nanowear experiments were carried out using diamond tip under the sliding conditions of *F*_n_ = 5 μN, *v* = 2 μm/s, *N* = 100, and RH = 40%. Insets display the AFM images of wear tracks.

Considering that the mechanical properties (e.g., elastic modulus and hardness) are the direct factors to determine the material removal behavior in general, the difference in mechanical properties between GaN-HF and GaN-AR surfaces should be verified. Detecting the intrinsic elastic modulus of oxidized layers on GaN-AR samples with a thickness of no more than 4 nm using a sharp Berkovich nanoindenter is a challenge due to the inevitable influence from monocrystalline GaN substrate. To minimize this influence and obtain a more accurate result, a spherical diamond nanoindenter with a radius of ~1 μm was used to measure the elastic modulus. The maximum penetrated depth was preset to 5 nm. Furthermore, nanoscratching tests were performed on GaN-HF and GaN-AR surfaces using a conic diamond probe with *R* ≈ 20 nm and *k* ≈ 100 N/m. Since the GaN/diamond pair is chemically inert, the interfacial mechanochemistry during the tribological process can be ruled out. In this case, the material removal is ascribed to the mechanical action under the contact pressure beyond the plastic yield of GaN material. Figure 4 displays the correlation of elastic modulus *E*_r_ and the removal volume *V* on GaN-HF and GaN-AR surfaces. The results indicate that the GaN-HF sample exhibits a higher elastic modulus (~320 GPa) than the GaN-AR sample (~280 GPa). The same as the previous studies (Xiao et al., 2017b), the softer surface shows weaker mechanical removal resistance and higher material removal volume under the same loading conditions. The mechanical removal induced by the diamond tip showed a similar trend to the mechanochemical removal by the Al_2_O_3_ microsphere (Figure 2), that is, the GaN-AR sample demonstrated a severer material removal than the GaN-HF sample. The higher removal rate of GaN-AR should be related to the weakened mechanical properties due to the natural oxidation reactions with water and oxygen molecules on the GaN sample surface.

Plastic deformation and flow normally occur after material yield (Johnson, 1987). Based on the relationship between the principal shear stress τ_c_ and maximum contact pressure *P*_max_, τ_c_ = 0.31*P*_max_ (Johnson, 1987), and the Tresca yield criterion, (τ_c_)_max_ ≤ 0.5σ_y_ where σ_y_ is the yield stress of GaN (15 GPa) (Nowak et al., 1999), the critical contact pressure *P*_y_ for the initial yield of GaN can be estimated as ~20 GPa using the following equation (Bhushan, 2013):

(1) $$\begin{array}{r} P_{\text{y}}=1/0.31{\left(\tau_{\text{c}}\right)}_{\text{max}}\;\leq \;1.61\sigma_{\text{y}}. \end{array}$$

This value is close to the hardness of GaN material (Hernández et al., 2020). The contact pressure at GaN-AR/Al_2_O_3_ interface can be evaluated using the DMT model (Schwarz, 2003),

(2) $$\begin{array}{r} P_{\max}=\frac{3}{2\pi }{\left[\frac{K^{2}}{R^{2}}\left(F_{n}+F_{a}\right)\right]}^{1/3}. \end{array}$$

Here, the combined elastic modulus *K* can be expressed by, *K* = 4/[3(1–$v_{1}^{2}$)/*E*_1_ + 3(1–$v_{2}^{2}$)/*E*_2_], where *E*_1_ and *E*_2_ are the elastic moduli of microsphere and substrate, *v*_1_ and *v*_2_ are the Poisson's ratio of Al_2_O_3_ microsphere and GaN substrate, respectively. It is known that the Poisson ratio of monocrystalline GaN and Al_2_O_3_ material is 0.25–0.35 (Fujikane et al., 2008). When the nanowear tests were conducted on GaN-AR substrate using Al_2_O_3_ tip under normal load of 4 μN (Figure 2) and *F*_a_ is the adhesion fore of ~0.1 μN, the contact pressure was estimated at ~1.5 GPa, which is slightly lower than that for the GaN-HF sample (1.6 GPa). Thus, the same as the GaN-HF surface, the sliding process on the GaN-AR surface shown in Figure 2B is also under the elastic contact region and the mechanical removal is not the dominant component in the material removal. Although here the mechanochemical removal dominates the material removal behavior, it does not mean that mechanical removal is completely ineffective.

### Influencing Mechanism of Oxide Layer on the Mechanochemical Removal of GaN Surface

**Figure 5:**
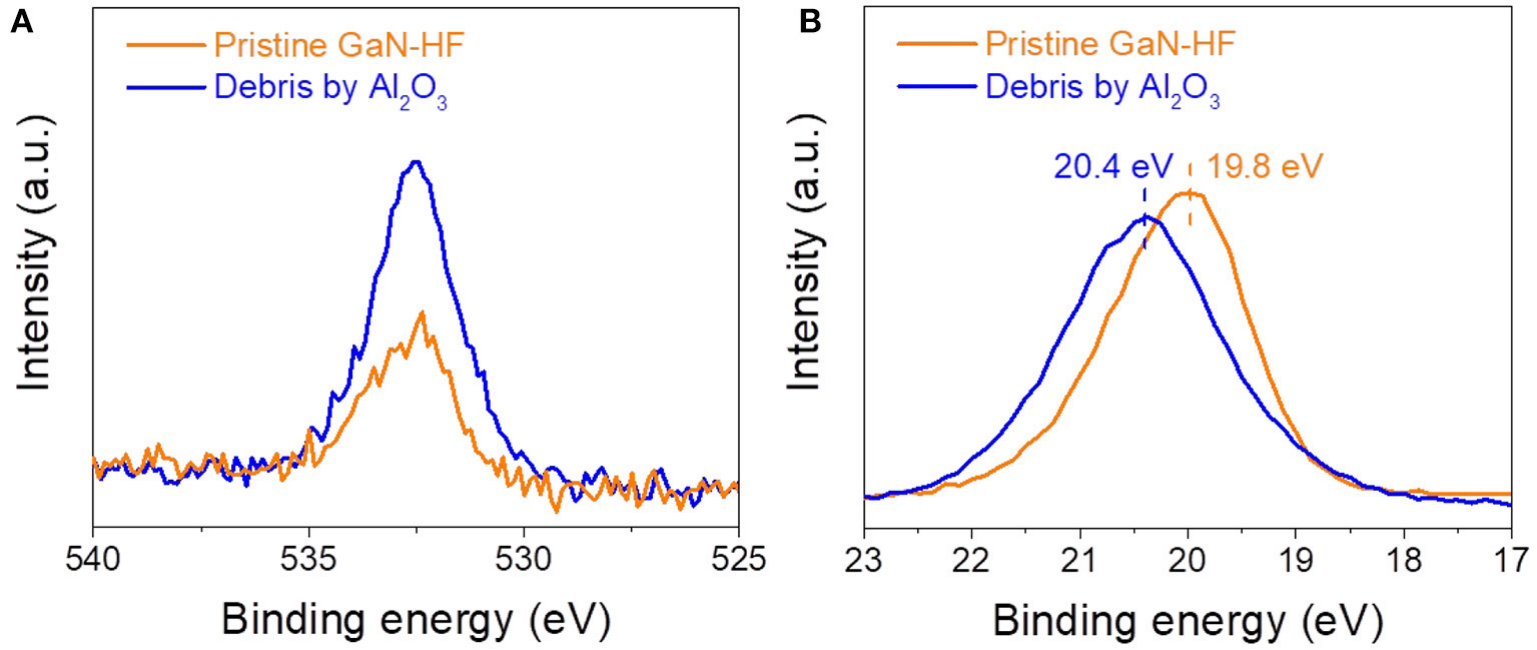
**(A)** O 1s and **(B)** Ga 3d SAXPS spectra of the wear debris produced on the GaN-HF surface and the pristine GaN-HF surface.

The above results showed that by combining chemically active counter-surface and external mechanical stress, material removal can occur on GaN surface due to the mechanochemical reactions in humid air under purely elastic contact situation, where the material removal does not introduce the lattice damage into the subsurface (Guo et al., 2021a). Since the strong oxygen signal of the oxide layer on the GaN-AR surface will cover the signal of wear debris completely, the chemical states of wear debris on GaN-HF substrate were characterized using SAXPS to further confirm the mechanism of material-removal caused by Al_2_O_3_ counter-surface. Given that the wear track produced using the Al_2_O_3_ tip cannot be detected due to the limited spatial resolution, the SAXPS detecting sample was prepared with a bigger Al_2_O_3_ sphere with a radius of ~3 mm. To suppress the mechanical damage of the GaN-HF substrate, the normal load was controlled at 0.5 N, and the corresponding contact pressure was 1.05 GPa. Figure 5 shows the O 1s and Ga 3d SAXPS spectra for the wear debris produced on the GaN-HF surface by sliding the Al_2_O_3_ sphere at 40% RH. As a reference, the O 1s and Ga 3d XPS spectra of the pristine GaN-HF surface were also plotted. The wear debris exhibited a more intense O 1s peak structure than that of the pristine GaN-HF surface. The detected O 1s signal on the pristine surface may be attributed to the inevitable oxide adsorption and residual oxygen in the XPS chamber. Wear debris with higher O 1s intensity suggested that the shear-assisted oxidation reactions were accompanied during the material removal process and the wear debris with the Ga_x_O_y_-like substance was the chemical product of oxidation reactions. Accordingly, the pristine GaN-HF surface has a narrow Ga 3d spectrum at 19.8 eV, whereas a broader peak (at 20.2 eV) was observed in the wear debris that was produced using the Al_2_O_3_ sphere (Zeng et al., 2018b). The broad peak of Ga 3d and a peak shift (leftward) of the Ga 3d spectra of the wear debris using Al_2_O_3_ sphere may be due to the heterogeneous structure comprising Ga-O and Ga-N bonds (Guo et al., 2019). Combined with the previous studies, the chemically active Al_2_O_3_ microsphere may promote the atomic attrition of GaN-HF substrate by forming bonding bridges of Al–O–Ga in watery environments (Zeng et al., 2018a,b) during the slight rubbing process. We hypothesized that the strained stress and mechanical energy were propagated to the GaN-HF substrate through the interfacial bonding bridges, resulting in a decreased activated energy of water-involved bonding rupture of the GaN crystal structure.

**Figure 6:**
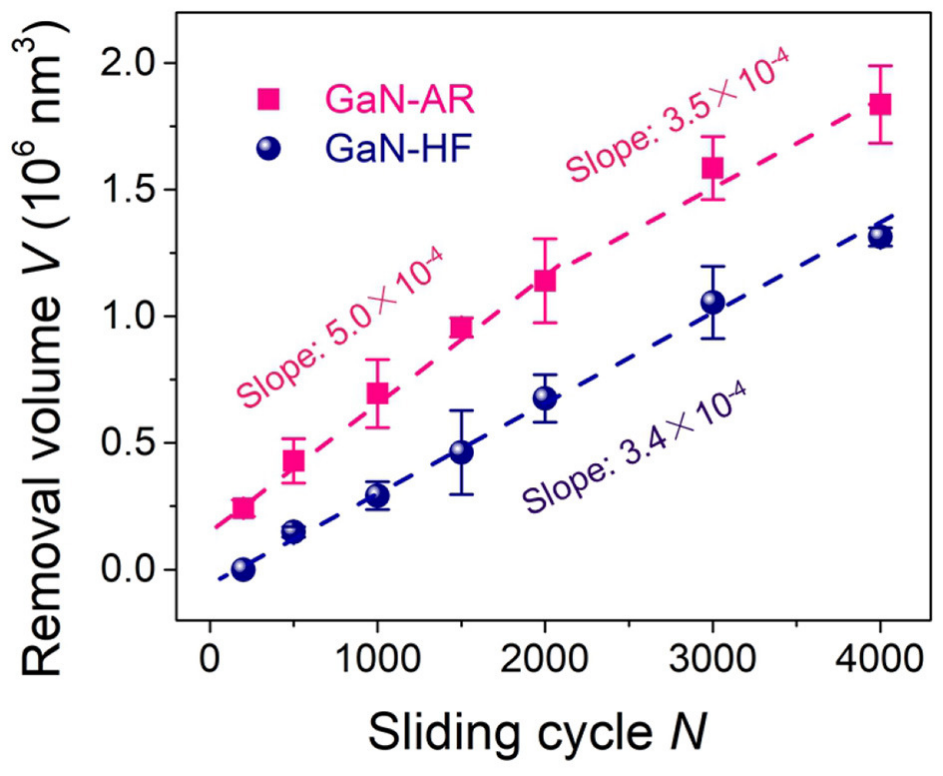
Removal volume vs. sliding cycle for GaN-HF and GaN-AR surfaces produced by sliding Al_2_O_3_ microsphere under the conditions of *F*_n_ = 4 μN, *N* = 2000, *v* = 2 μm/s, and RH = 60%.

The aforementioned AES results (Figure 1B) have proved that the oxide layer on the GaN-AR surface is almost removed by HF etching, thus, the tribological interface basically remains unchanged with the increased wear depth when the Al_2_O_3_ microsphere rubbing the crystal GaN-HF surface. By contrast, the GaN-AR sample can be taken as a double-layer structural substrate, that is, the monocrystalline GaN layer at the bottom is covered with an oxide layer with gradually increasing oxygen content (bottom-up). Such graded oxide layer had an evident influence on the mechanochemical reaction at the GaN–Al_2_O_3_ interface (Figure 2). To investigate the evolution of material removal rate with the increasing wear depth, the nanowear tests were performed on the GaN-HF and GaN-AR surfaces using Al_2_O_3_ microsphere under different reciprocating sliding cycles, as shown in Figure 6. The material-removal rate was obtained according to the linear slope by fitting the curve of the material removal volume vs. sliding cycle (reaction time). Assuming that the gradient change of oxygen content in the oxide layer does not affect the material removal rate or the difference caused by the gradient-changed oxygen content cannot be distinguished within the experimental accuracy of this study, the variation of the material removal volume of the GaN-AR surface with the sliding cycle can be divided into two linear segments with the turning point at the 2000 cycles. The values of the linear slope before and after 2,000 cycles are 5.0 × 10^2^ nm^3^/cycle and 3.5 × 10^2^ nm^3^/cycle, respectively. After about 2,000 sliding cycles of wear, the oxide layer in the contact area was almost removed and the Al_2_O_3_ tip began to directly rub the monocrystalline GaN substrate, which is also consistent with the AES measurement of oxide layer thickness (Figure 1B), and the results of nanowear experiments (Figure 2B). It is indicated that the material removal rate of oxidized GaN is higher than that of monocrystalline GaN. Moreover, in the case of GaN-HF substrate, a linear relationship between the removal volume and sliding cycles covered the entire reciprocating sliding process. The fitted slope value was close to that in the second segment for the GaN-AR sample, at this moment such value was the material-removal rate of the pure monocrystalline GaN.

The above results suggest that the material removal of GaN-AR and GaN-HF surfaces under the elastic contact region is dominated by the interfacial mechanochemical reaction, which can be described as the shear-induced or stimulated chemical reaction of the GaN substrate with the assistance of water molecules. Such reactions can hardly occur under pure thermodynamics or mechanics but can be activated by external mechanical energy. Thus, the mechanochemical activation energy (*E*_a_) at the GaN–Al_2_O_3_ interface is considerably larger than the thermal energy (~*RT*, where *R* is the gas constant with a value of 8.314 J/K, and *T* is equal to 300 K at room temperature) (Stocker et al., 1998). Under the shear force of the Al_2_O_3_ microsphere, *E*_a_ can be physically lowered by the external mechanical energy, and the reduction magnitude in *E*_a_ is represented as *E*_m_ (Beyer and Clausen-Schaumann, 2005; Peguiron et al., 2016). Furthermore, the effective activation energy *E*_eff_ in the chemical-reaction coordinate can be written as *E*_eff_ = *E*_a_-*E*_m_ (Jacobs and Carpick, 2013; Gosvami et al., 2015; Chen et al., 2017; Yeon et al., 2017). Hence, the normalized mechanochemical removal rate (*k*) of GaN material against Al_2_O_3_ microsphere can be theoretically modeled with a mechanically modified Arrhenius-type kinetic model: (Beyer, 2000; Gotsmann and Lantz, 2008; Spikes and Tysoe, 2015; Tysoe, 2017; Spikes, 2018).

(3) $$\begin{array}{r} k=A\cdot \exp\frac{-\left(E_{a}-E_{m}\right)}{k_{b}T}, \end{array}$$

where *k*_b_ is the Boltzmann constant (1.38 × 10^−23^ J/K), *A* is the pre-exponential factor, and *T* is the temperature at the tribological interface and can be idealized to be unchanged due to the ultralow sliding speed during the nanoscratching process (Xiao et al., 2019). Based on the dimensionality argument, *E*_m_ can be expressed as the work that is done by the applied load *F* and shear stress σ: *E*_m_ = *F*·Δx^*^ = σ·ΔV^*^. Here, Δx^*^ can be considered to be the minimum amount of bond-length change needed to reach the transition state. In laboratory experiments, it would be difficult to control the force applied to individual atoms or molecules, and thus shear stress (σ) should be a more appropriate term to consider. Then, ΔV^*^ can be described to the volume difference between the equilibrium and transition states of targeted chemical bond during the shear-induced mechanochemical reaction (Jacobs and Carpick, 2013; Gosvami et al., 2015; Yeon et al., 2017). Then σ can be determined by the linear relationship with the contact pressure *P*: σ = μ·*P* + σ_0_, where μ is the friction coefficient of the tribological interface, and σ_0_ is nonzero term depending on the interfacial attractive interaction (Samoilov et al., 2007; Arnell, 2010). Replacing *E*_*m*_ with (μ·*P* + σ_0_)·ΔV^*^ and taking the logarithm of both sides, equation (3) can be extended as follows:

(4) $$\begin{array}{r} \ln\;\left(k\right)=\left(\text{ln}A-\frac{E_{a}}{k_{b}T}+\frac{\sigma_{0}\Delta V^{*}}{k_{b}T}\right)+\frac{\Delta V^{*}\mu }{k_{b}T}P \end{array}$$

**Figure 7:**
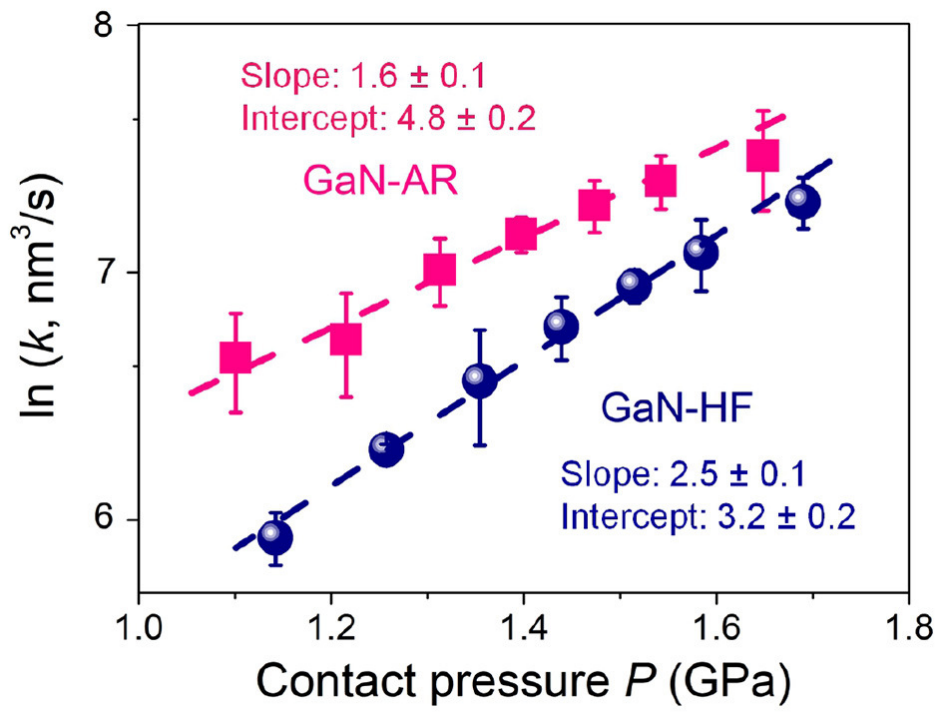
Semi-log plot of the contact pressure-dependent mechanochemical removal rate *k* of GaN-HF and GaN-AR surfaces rubbed by Al_2_O_3_ microsphere under the conditions of *N* = 2000, *v* = 2 μm/s, and RH = 60%.

To reveal the mechanical activation in the mechanochemical reaction, load (contact pressure) dependence of reaction/removal rate of the GaN-HF and GaN-AR substrates against Al_2_O_3_ microsphere were discussed. The wear process of the GaN-AR surface was controlled strictly in the upper oxide layer (wear depth < 4 nm) to eliminate the substrate effect on the subsequent mechanism analysis of mechanochemical reactions. Figure 7 plots the relationship between *ln*(*k*) and *P* following the mechanically modified Arrhenius-type kinetic model. Based on the linear regression of *ln*(*k*) on *P*, the slope $\frac{\Delta V^{*}\mu }{k_{b}T}$ (GPa^−1^) was fitted as 2.5 ± 0.1 and 1.6 ± 0.1 for the GaN-HF and GaN-AR surface, providing the ΔV^*^ as 12.8 ± 0.5 and 8.2 ± 0.5 Å^3^, respectively. These deviations were calculated by the standard error of the linear regression. The intercept $\text{ln}A-\frac{E_{a}}{k_{b}T}+\frac{\sigma_{0}\Delta V^{*}}{k_{b}T}$ [ln(nm^3^/s)] for GaN-HF and GaN-AR were fitted as 3.2 ± 0.2 and 4.8 ± 0.2, respectively. Although the absolute value of *E*_a_ cannot be obtained without determining *A*, this expression physically represents the self-driven force of the mechanochemical reactions that occurs at the tribological interface when the external mechanical interaction is negligible. Based on the computational results regarding the physical meaning of the ΔV^*^, the value is described as the minimum amount of change in molecular volume to initiate chemical reactions of that specific molecule by shear and compressive stress, reflecting the degree of the required shear-induced structural deformation to rupture Ga–N or Ga–O–N bond via mechanochemical reactions (Yeon et al., 2017; Guo et al., 2021b). The above results demonstrate that less external mechanical activation is required to initiate the mechanochemical atomic attrition for oxidized GaN surface compared with the pristine GaN surface without oxide layer. Accordingly, an evident reduction in intercept (negatively correlated with *E*_a_) illuminates the stronger mechanochemical removal resistance on the GaN-HF surface. Thus, oxidized GaN-AR can easily undergo a bonding breakage process upon small structure distortion or stretching from its equilibrium structure due to the external mechanical forces at the sliding interface, giving the smaller ΔV^*^ than those of the GaN-HF surface. These findings in this study can advance the understanding of the influencing mechanism of the surface oxidation in mechanochemical reaction at the GaN–Al_2_O_3_ interface and provide fundamental knowledge to improve the machining efficiency in the ultra-precision surface manufacturing of GaN material. The mechanochemistry-associated material-removal mechanism at the GaN–Al_2_O_3_ interface might be applicable to other oxide counter surfaces, such as SiO_2_, CeO_2_, etc., which are also commonly used as the abrasive particles in CMP. Moreover, the effects of chemical activity and mechanical properties of these oxide counter-surfaces on the mechanochemical material-removal will be further discussed in the future to provide useful theoretical reference for high-efficiency and non-destructive CMP technique toward monocrystalline GaN.

### Conclusions

By using AFM, mechanochemical removal behaviors on the GaN-HF and GaN-AR surfaces against Al_2_O_3_ microsphere were investigated with a humidity range of 3–80%. Under a purely elastic contact region, evident material removal can occur on GaN-HF and GaN-AR surfaces with the assistance of the interfacial mechanochemical reactions. Experimental results show that the mechanochemical reactions at the GaN–Al_2_O_3_ interface can be facilitated by the topmost oxidized surface structure over the entire humidity range. Analyzing the mechanochemical reactions with the Arrhenius-type kinetic model and DMT contact mechanics reveal that the critical activation volume and activation barrier can semi-quantitatively describe the external mechanical activation for the mechanochemical reactions Less external mechanical activation is required to initiate the mechanochemical atomic attrition on the GaN-AR surface compared with that on the GaN-HF surface. These findings can gain new insight into the underlying mechanism of mechanochemical reactions at the GaN–Al_2_O_3_ interface and provide fundamental knowledge for the oxidation-assisted ultra-precision surface machining of GaN.

## Data Availability Statement

The original contributions presented in the study are included in the article/supplementary material, further inquiries can be directed to the corresponding author.

## Author Contributions

JGu: investigation, conceptualization, writing–original draft, and funding acquisition. CX: investigation, conceptualization, data curation, and writing–review and editing. JGa and JL: investigation and data curation. LC: writing–review and editing. LQ: supervision and resources. All authors have read and approved the manuscript.

## Conflict of Interest

The authors declare that the research was conducted in the absence of any commercial or financial relationships that could be construed as a potential conflict of interest.
